# Heavy Metal Tolerance Trend in Extended-Spectrum β-Lactamase Encoding Strains Recovered from Food Samples

**DOI:** 10.3390/ijerph18094718

**Published:** 2021-04-28

**Authors:** Kashaf Junaid, Hasan Ejaz, Iram Asim, Sonia Younas, Humaira Yasmeen, Abualgasim Elgaili Abdalla, Khalid Omer Abdalla Abosalif, Ayman Ali Mohammed Alameen, Naveed Ahmad, Syed Nasir Abbas Bukhari, Abdul Rehman

**Affiliations:** 1 Department of Clinical Laboratory Sciences, College of Applied Medical Sciences, Jouf University, Skaka 72388, Saudi Arabia; hetariq@ju.edu.sa (H.E.); aealseddig@ju.edu.sa (A.E.A.); koabosalif@ju.edu.sa (K.O.A.A.); aaalameen@ju.edu.sa (A.A.M.A.); 2 Department of Microbiology and Molecular Genetics, The Women University, Multan 66000, Pakistan; Iramasim.mmg@gmail.com (I.A.); humaira.6127@wum.edu.pk (H.Y.); 3 Department of Pathology, Tehsil Headquarter Hospital Kamoke, Kamoke 50661, Pakistan; soniamicro02@gmail.com; 4 Department of Pharmaceutics, College of Pharmacy, Jouf University, Sakaka 72388, Saudi Arabia; nakahmad@ju.edu.sa; 5 Department of Pharmaceutical Chemistry, College of Pharmacy, Jouf University, Sakaka 72388, Saudi Arabia; sbukhari@ju.edu.sa; 6 Department of Microbiology and Molecular Genetics, University of the Punjab, Lahore 54590, Pakistan; rehman_mmg@yahoo.com

**Keywords:** processed food, food contaminants, heavy metals, beta-lactamase, ESBL

## Abstract

This study evaluates bacteriological profiles in ready-to-eat (RTE) foods and assesses antibiotic resistance, extended-spectrum β-lactamase (ESBL) production by gram-negative bacteria, and heavy metal tolerance. In total, 436 retail food samples were collected and cultured. The isolates were screened for ESBL production and molecular detection of ESBL-encoding genes. Furthermore, all isolates were evaluated for heavy metal tolerance. From 352 culture-positive samples, 406 g-negative bacteria were identified. Raw food samples were more often contaminated than refined food (84.71% vs. 76.32%). The predominant isolates were *Klebsiella pneumoniae* (*n* = 76), *Enterobacter cloacae* (*n* = 58), and *Escherichia coli* (*n* = 56). Overall, the percentage of ESBL producers was higher in raw food samples, although higher occurrences of ESBL-producing *E. coli* (*p* = 0.01) and *Pseudomonas aeruginosa* (*p* = 0.02) were observed in processed food samples. However, the prevalence of ESBL-producing *Citrobacter freundii* in raw food samples was high (*p* = 0.03). Among the isolates, 55% were *bla*_CTX-M_, 26% were *bla*_SHV_, and 19% were *bla*_TEM_. Notably, heavy metal resistance was highly prevalent in ESBL producers. These findings demonstrate that retail food samples are exposed to contaminants including antibiotics and heavy metals, endangering consumers.

## 1. Introduction

Microbiological risks associated with consuming ready-to-eat (RTE) food have recently become more common. Numerous outbreaks of pathogenic microorganisms demonstrate that food items are potential carriers of microorganisms that cause foodborne diseases [1]. RTE food contains pathogenic and nonpathogenic microbial flora, particularly gram-negative bacteria such as *Escherichia*, *Vibrio*, *Shigella*, *Salmonella*, *Campylobacter*, and *Klebsiella* species [2,3].

The incidence of foodborne diseases is high in developing countries. However, no region of the world is devoid of foodborne diseases [4]. Several studies have found extended-spectrum β-lactamase (ESBL)-producing organisms in fruits and vegetables in retail [1,5]. Recently, a study reported the prevalence of ESBL on bean sprouts and other vegetables in South Korea and the Netherlands [6]. Through the application of animal-product biofertilizers, edible plants and fruits may become infected with antibiotic-resistant and pathogenic bacteria during development and serve as vectors of transmission to humans [7].

Moreover, a significant association between food handlers and foodborne outbreaks of ESBL-producing *Enterobacteriaceae* was reported in Spain [8]. In another study, a nosocomial outbreak of ESBL-producing *K. pneumoniae* was also reported and the drug resistant strain was transmitted through food [9]. Additionally, a study in Kenya determined that fecal ESBL contaminants in food (3%) originated from food handlers [10].

The accessibility of inexpensive RTE food comes at the cost of quality in low-income populations [11]. Unhygienic handling of food from production through delivery is often seen in Pakistan. There are many opportunities for multi-resistant foodborne pathogen contamination of retail food items.

The association between heavy metal-tolerant bacteria in food items and antibacterial drug resistance is insufficiently studied. Antibiotic-resistant bacteria are enriched in metal-contaminated environments, and genes conferring co-selection for heavy metal- and antibiotic-tolerance are frequently found together in clinical isolates [12,13]. The association between antibiotic-resistant bacteria and raw and processed foods is also poorly understood. This study evaluated the prevalence of ESBL-producing gram-negative isolates in retail food samples. In addition, isolates were compared to determine the association between ESBL production and heavy metal tolerance.

## 2. Materials and Methods

### 2.1. Study Design and Sample Collection

**Figure 1:**
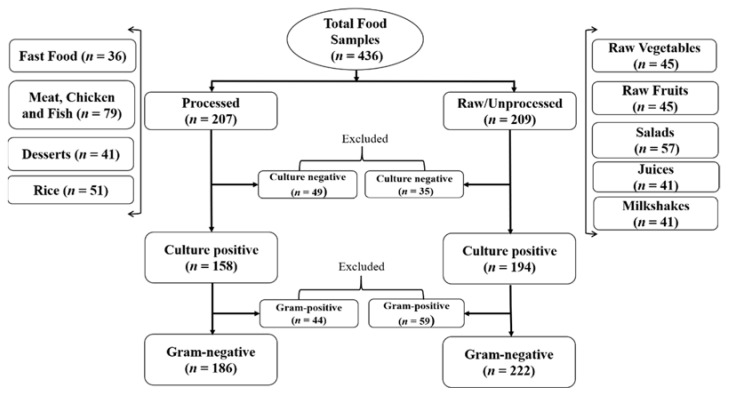
Types of food samples collected and processed in this study (*n* = 436).

In this study, 436 different food samples were collected from seven Punjab districts (Bahawalpur, Jahania, Multan, Budhla Sant, Dunyapur, Jhang, and Khanewal) in Pakistan. All food samples were collected from street vendors, local markets, hospitals, and university cafeterias. In total, 207 processed food samples (fast food and cooked meat, chicken, fish, rice, and desserts) and 229 raw or unprocessed food samples (raw vegetables/fruits, salads, juices, and milkshakes) were collected. The details of all included samples and the sample flow are presented in Figure 1. The samples were aseptically collected, i.e., solid food was placed in sterile bags, and liquid food was collected in sterile screw-capped bottles. All samples were kept at 4 °C until further processing.

### 2.2. Specimen Collection and Processing

For processing, 250 g of solid food samples and 10 mL of liquid samples were collected for microbiological analysis. Solid samples were enriched by crushing with a mortar and pestle. Subsequently, 25 g of the crushed specimen was homogenized with 225 mL of peptone water broth. The homogenized mixture was shaken (160 rpm) for 1–2 h and then further processed.

### 2.3. Isolation and Characterization of Gram-Negative Bacteria

Enriched cultures and liquid samples were streaked onto MacConkey agar, and the plates were incubated at 37 °C overnight. Presumptive identification of isolates was performed based on colony morphology, Gram staining, and biochemical tests [14,15]. Only gram-negative isolates were further subjected to biochemical identification. For additional confirmation, the API 20E and 20NE tests were performed.

### 2.4. Antimicrobial Sensitivity Testing

In vitro antimicrobial drug sensitivity was evaluated using 18 drugs recommended for use against gram-negative bacteria. Each isolate’s colonies were aseptically emulsified in normal saline to produce a suspension meeting to 0.5 McFarland standards. The suspended isolate was streaked onto a Mueller Hinton agar culture plate, and antibiotic discs were placed on the culture plate surface. Culture plates were incubated at 37 °C for 18–24 h, and each organism was reported as sensitive or resistant to each antibiotic according to the zone of inhibition. The following antibiotics were used in this study: aztreonam (30 µg), amikacin (30 µg), gentamicin (10 µg), cefuroxime (30 µg), cefoxitin (30 µg), ceftriaxone (30 µg), ceftazidime (30 µg), cefotaxime (30 µg), cefepime (30 µg), ciprofloxacin (5 µg), levofloxacin (5 µg), imipenem (10 µg), meropenem (10 µg), piperacillin-tazobactam (100/10 µg), colistin (10 µg), co-trimoxazole (1.25/23.75 µg), and tigecycline (15 µg). To ensure the reliability of in vitro antibacterial sensitivity testing, ATCC quality control (QC) strains of *K. pneumoniae* (700603, ESBL-positive) and *E. coli* (25922, ESBL-negative) were used, as recommended by CLSI guidelines [16].

### 2.5. Phenotypic Characterization of ESBL Producers

According to accepted guidelines, the double-disc synergy test and the combined-disc test were performed on Mueller Hinton agar plates to characterize the phenotypic characteristics of ESBL-producing strains. Cephalosporin and clavulanate antibiotics were used in both tests [17].

### 2.6. Molecular Characterization of MCR Genes

Genes encoding ESBL were amplified from all phenotypically characterized ESBL producers. Previously reported primers for *bla*_SHV_, *bla*_TEM_, and *bla*_CTX-M_ were used, and the PCR conditions were optimized [18]. PCR conditions were followed as: initial denaturation at 95 °C for 3 min; 30 cycles of 15 s at 95 °C, 56 °C for 35 s and 72 °C for 1 min; finally, a 7 min extension step at 72 °C. The amplified products were visualized by agarose gel electrophoresis, and product sizes were determined using a DNA ladder (100 bp). A UV transilluminator and gel documentation system were used to collect digital images of the PCR products. The PCR products were submitted to reverse and forward gene sequencing and analyzed using FinchTV v. 1.4 (Geospiza, Inc., Seattle, WA, USA). To identify *bla* gene variants, programs such as BlastN, BlastP (NCBI), and ExPASy (SIB Group) were used for nucleotide, amino acid, and translational analyses.

### 2.7. Resistance to Heavy Metals

According to a previously published procedure, the heavy metal tolerance of each isolate was examined [19,20] using the heavy metal salts molybdenum oxide, chromium chloride, arsenic chloride, and cobalt chloride. Briefly, each isolate was assessed on agar plates containing increasing concentrations of each heavy metal salt. The heavy metal concentration ranged between 250 μg/100 mL and 1500 μg/100 mL. Each plate with a higher heavy metal concentration was inoculated with the isolate from the plate with the previous concentration, and the plates were incubated at 37 °C for 24 h and examined for growth.

### 2.8. Statistical Analysis

The data were analyzed using SPSS version 24.0 (Armonk, NY, USA) and GraphPad Prism 6.0 (San Diego, CA, USA). Descriptive analysis was performed to calculate the frequencies and percentages of each variable. The chi-square test and regression analysis were used for inferential statistics.

## 3. Results

The results revealed that of 436 food samples collected in this study, 352 (80.73%) were culture-positive. Raw food samples were more often contaminated than processed food samples (84.71% vs. 76.32%). The average CFU count for raw food samples was 7.4 × 10^5,^ and 5.6 × 10^4^ for processed food samples was observed. From the 352 culture-positive samples, 408 g-negative bacterial isolates (186 from processed food samples and 222 from raw food samples) were obtained and identified. Among the isolates, the most abundant organisms were *K. pneumoniae* (*n* = 76), *E. cloacae* (*n* = 58), *E. coli* (*n* =56), *S. typhimurium* (*n* = 43), *Serratia marcescens* (*n* = 38), *S. enteritidis* (*n* = 35), *C. freundii* (*n* = 29), *Y. enterocolitica* (*n* = 21), *P. aeruginosa* (*n* = 19), *Shigella* spp. (*n* = 17), and *P. mirabilis* (*n* = 16).

### 3.1. Association of Isolates with the Food Sample Type

The most prevalent isolate from the raw food sample was *K. pneumoniae* (19.82%), and from processed food samples, it was *E. coli* (20.43%). *E. coli* (20.43%, *p* < 0.01) and *P. aeruginosa* (7.53%, *p* = 0.01) were associated with processed food. In contrast, *S. typhimurium* (14.4%, *p* = 0.01) and *S. marcescens* (12.16%, *p* = 0.03) were potentially associated with raw food. No marked differences in prevalence according to the food sample type were observed for the other organisms (Table 1).

### 3.2. Antimicrobial Sensitivity Pattern

The overall results of antibacterial drug resistance testing against 18 drugs showed that most isolates were resistant to ampicillin (90%). The highest cephalosporin antibiotic resistance was observed for cefuroxime (51%), and the lowest resistance was observed for cefoxitin and cefepime (22%). The carbapenem and quinolone resistance percentages were similar (17% and 18%, respectively). This study revealed minimal resistance to tigecycline (6%) and colistin (2%). The detailed antibacterial drug resistance profiles of the individual bacterial species are presented in Table 2.

### 3.3. Detection of ESBL-Producing Organisms and Associations with Food Samples

Of the 408 isolates from food samples, 173 (42.4%) exhibited ESBL production, including 84 (48.5%) from processed food samples and 89 (51.5%) from raw food samples. The following isolates with resistant phenotypes were identified: *K. pneumoniae* (*n* = 39), *E. cloacae* (*n* = 16), *E. coli* (*n* = 36), *S. typhimurium* (*n* = 17), *Serratia marcescens* (*n* = 7), *S. enteritidis* (*n* = 11), *C. freundii* (n = 14), *Y. enterocolitica* (*n* = 5), *P. aeruginosa* (*n* = 14), *Shigella* spp. (*n* = 4), and *P. mirabilis* (*n* = 10).

We assessed the presence of ESBL-producing organisms relative to the food type. The results indicated a higher prevalence of ESBL-producing *E. coli* (*p* = 0.01, odds ratio (OR): 2.64 and 95% confidence interval (CI): 1.22–5.72) and *P. aeruginosa* (*p* = 0.02, OR: 4.43 and 95% CI: 1.19–12.60) in processed food samples. In raw food samples, the incidence of ESBL-producing *C. freundii* was high (*p* = 0.03, OR: 3.71 and 95% CI: 1.52–12.50). However, the other ESBL-producing organisms did not show any statistically significant associations with the food sample type in this study (Table 3).

### 3.4. Molecular Characterization of ESBL-Encoding Genes

**Figure 2:**
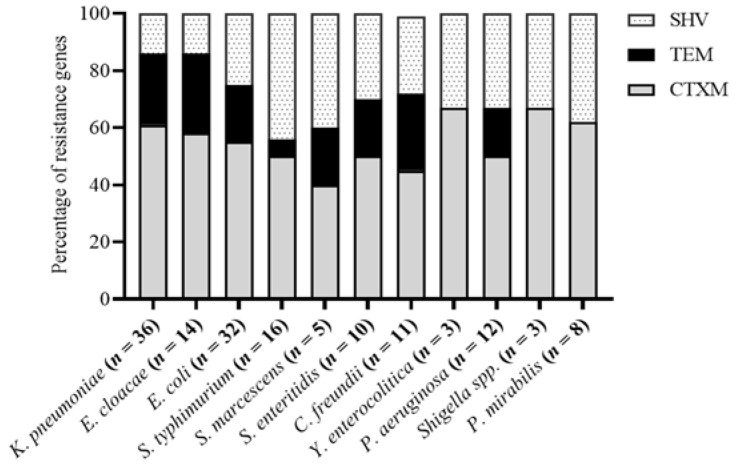
Genetic distribution of ESBL-encoding genes in the isolates (*n* = 150).

Of 173 isolates phenotypically characterized as ESBL-producing, 150 (86.71%) harbored ESBL-encoding genes. According to PCR results, *bla*_CTX-M_, *bla*_SHV_, and *bla*_TEM_ genes were detected in 83 (55%), 39 (26%), and 28 (19%) isolates, respectively. Among *K. pneumoniae* isolates, 61%, 25%, and 14% were positive for *bla*_CTX-M_, *bla*_TEM_, and *bla*_SHV_, respectively. Among *E. cloacae* isolates, 58%, 28%, and 14% harbored *bla*_CTX-M_, *bla*_TEM_, and *bla*_SHV_, respectively. A high percentage of *bla*_CTX-M_ was observed in all isolates. The frequency of *bla*_TEM_ was similar in *E. coli*, *S. marcescens*, and *S. enteritidis*. Similarly, the frequency of *bla*_SHV_ was the same in *S. enteritidis*, *Shigella* spp., *Y. enterocolitica*, and *P. aeruginosa*. The distribution of ESBL-encoding genes in each isolate is presented in Figure 2.

Moreover, the detailed distributions of gene variants in these isolates are presented in Table 4. The highest incidence was reported for *bla*_CTXM-1_ (56), followed by *bla*_SHV-12_ (35), *bla*_TEM-1_ (25), *bla*_CTXM-2_ (21), *bla*_CTXM-9_ (6), and *bla*_TEM-135_ (2), with only one isolate harboring *bla*_TEM-4_.

### 3.5. Heavy Metal Resistance

**Figure 3:**
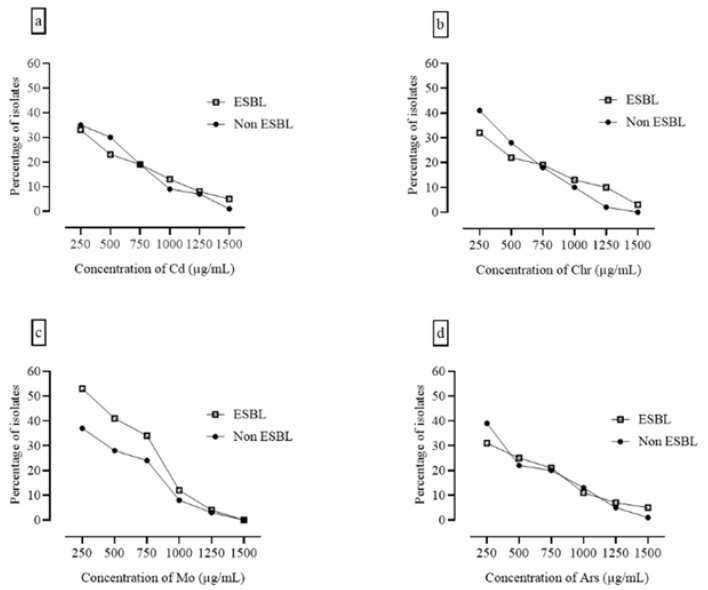
Number of heavy metal resistant isolates at different concentrations of heavy metals. As the concentration of heavy metal increased, the number of organisms decreased. (**a**) High resistance trend of ESBL producers against Cadmium. (**b**) High resistance trend of ESBL producers against Chromium. (**c**) High resistance trend of ESBL producers against Molybdinieum (**d**) High resistance trend of ESBL producers against Arsenic.

Further, all isolates were assessed for heavy metal resistance against four metals at increasing concentrations up to 1500 µg/mL. The results indicated potential heavy metal contamination in the RTE food samples. No clear differences were observed among heavy metal resistance patterns. However, a uniform decrease in the number of resistant isolates was observed with increasing heavy metal concentrations. Selective screening of heavy metal-resistant isolates showed that the isolates with maximal resistance were also ESBL producers. Figure 3 presents the numbers of bacterial isolates in the ESBL and non-ESBL groups that showed resistance against heavy metals at the indicated concentrations.

## 4. Discussion

Beta-lactamase-harboring gram-negative bacteria are no longer exclusively linked to the health care system. Therefore, investigating the possible threats to food safety and integrity posed by these bacteria has become increasingly important. The dissemination and expansion of antimicrobial resistance genes and resistant bacteria are neither confined to animals and humans nor by geographic boundaries. This study principally investigated the predominance of ESBL-producing gram-negative isolates in retail food samples. We processed 436 food samples, including raw and processed foods. Among these samples, 352 (80.73%) were culture-positive.

In total, 408 g-negative bacterial isolates were recovered from 352 positive cultures, of which 42.4% were ESBL producers. Our study indicated a 42.4% ESBL rate, which was higher than the 25.4% rate reported by Zurfluh et al. and lower than the 79.2% rate found by Richter et al. [21,22]. However, the ESBL proportion found in our study was higher than those reported in Vietnam [23] and Southwest Ethiopia [24], and lower than that reported in China [25]. The inconsistent prevalence rates may reflect differences in the study methodology, isolation technique, sample size and type, and collection process; thus, the comparison of results from different countries may not be feasible.

A high (51.5%) incidence of ESBL producers in raw foods (vegetables, fruits, salads, juice, and milk) was detected in our study, which was in agreement with the results of previous reports from South Africa and Pakistan [3,22]. These findings indicate that raw and fresh foods can propagate and expand ESBL and drug resistance genes to consumers, as previously reported [26,27]. Another critical finding is that manure of animal origin, sewage and sludge, pesticides, and crop wastewater pollute soil and crops, providing a source of antibiotic resistance.

We also observed prevalent ESBL production in processed food (48.5%), which indicates cross-contamination in the production process. Possible sources of contamination in processed food could include food handlers, food utensils, unsafe temperatures, and poor personal hygiene. A study in China reported more ESBL production in frozen food than in raw food, which is in contrast to our results [25].

The overwhelming majority of ESBL-producing bacterial isolates in our study were *K. pneumoniae* and *E. coli*, followed by *S. typhimurium*, *E. cloacae*, and *P. aeruginosa*, which was consistent with previous results in China and Vietnam [21,28]. Two additional studies reported *K. pneumoniae*, *E. coli*, *S. typhimurium*, *E. cloacae*, *Citrobacter*, and *P. aeruginosa* in a variety of food samples [29,30]. We found *K. pneumoniae* to be the predominant strain in both raw and processed foods, followed by *E. coli*. Two studies in China showed similar results, identifying *E. coli* as the most prevalent organism [28,31]. Interestingly, *S. typhimurium* was also found in raw (14.41%) and processed foods (5.91%), indicating poor hygiene and inadequate hand-washing practices, as this bacterium is spread from unwashed hands of infected food handlers via improper preparation and poor handling of foods, resulting in food contamination. Sofy et al. reported *S. typhimurium* as the second-most prevalent organism isolated from food samples [2].

The presence of *E.coli* and *P.aeruginosa* in processed food indicated that the food was either undercooked or subjected to post-cooking contamination, which may have occurred due to improper handling, prolonged storage, inappropriate chilling, or several other conditions. In comparison, the presence of *S. typhimurium* and *S. marcescens* in raw food may be acquired during processing as these bacteria are not naturally present in food items. Additionally, polluted water may be sprayed on the vegetables or vegetables may be grown on polluted soil with contaminated irrigation water. Local sanitary conditions may exist during the processing, post-production, transportation, storage, and retail of fresh vegetables [32,33].

Previous studies mainly indicated the presence of environmental bacteria harboring chromosome-mediated beta-lactamase resistance genes, but our study and other recent studies produced different results. A high prevalence of pathogenic bacteria such as *Salmonella* and some opportunistic bacteria, including *E. coli*, *K. pneumoniae*, *Citrobacter* spp. and *Enterobacter* spp., which not only cause community-acquired human infections but also carry plasmid-mediated resistance genes [34,35,36] was found.

Generally, *bla*_TEM_, *bla*_SHV_, and *bla*_CTX-M_ are common beta-lactamase genes found broadly. However, CTX-M-β-lactamase is the most prevalently reported ESBL worldwide [37,38]. Our study demonstrated the distributions of various ESBL genes, with *bla*_CTX-M_ showing high prevalence, which is in agreement with previous results [37,38]. According to PCR results, 83 (55%) *bla*_CTX-M_, 39 (26%) *bla*_SHV_, and 28 (19%) *bla*_TEM_ genes were detected in our study, corresponding to previous findings [25]. We identified *bla*_CTX-M-1_ as the dominant variant among *K. pneumoniae*, *E. cloacae*, and *E. coli*, while *bla*_CTX-M-15_ was the most prevalent among *E. coli* and *K. pneumoniae* isolates in some previous reports [21,22].

High antimicrobial resistance against various groups of antibiotics was observed in our study, indicating a multidrug-resistance phenotype. Analysis of the antibiotic susceptibility profile revealed that most isolates were resistant to ampicillin (90%), which is consistent with previous reporting in China [25]. We found high resistance to the cephalosporin antibiotic cefuroxime (51%), and similar results were reported in a previous study [39]. For carbapenem, a moderate resistance rate was found, which is in agreement with previous reporting in China, indicating misuse of carbapenem drugs. This study revealed minimal resistance to tigecycline (6%) and colistin (2%) [13,40].

Heavy metals also exert antimicrobial effects, and some hospitals use metallic copper-coated surfaces to reduce the risk of nosocomial contamination [39]. Co-selection of antimicrobial and heavy metal resistance in bacteria has greatly affected the efficacy of available drugs and therapies for infectious diseases. To understand the role of heavy metals, we assessed all isolates against four heavy metals with increasing concentrations up to 1500 µg/mL. The isolates indicated potential heavy metal contamination in RTE food samples. We found no clear differences between resistance patterns. However, selective screening of heavy metal-resistant isolates showed that those with maximal resistance were also ESBL producers. Many studies have reported the potential threat to the environment and foodstuff posed by heavy metals [41,42].

The presence of arsenic-, chromium-, molybdenum-, cobalt-, and mercury-resistant isolates in food samples can be attributed to the accumulation of high metal salt concentrations in soil and water, resulting in contamination with heavy metals such as arsenic, and the use of metal utensils. Metal resistance trends varied considerably depending on the sample site and sample type. There was a significant correlation between antibiotic resistance and metal resistance, indicating that these properties are linked. This study revealed high bacterial loads in foods readily and regularly consumed. The results established the incidence of ESBL-producing and heavy metal-resistant pathogenic bacteria in RTE foods sold locally in Punjab, Pakistan.

The present study highlighted several areas for improvement. The implementation of hygienic practices is mandatory throughout the food cycle, starting from manufacturing, processing, and cooking. Fruits and vegetables are irrigated with sewage in several fields in Pakistan. The use of contaminated water in washing the food imposes another risk of contamination. The use of contaminated hands and water in milking leads to milk contamination. Boiled or un-contaminated water should be used to water the vegetables and fruits. The implementation of vigilant hygienic measures on each of the steps mentioned above from the vendors and the consumers could help to reduce the risk of pathogen transmission. Effective surveillance and monitoring of food items and establishing and imposing regulations will ultimately reduce contaminants in the food samples.

To our knowledge, this is the first report of the high occurrence of coexisting ESBL production and heavy metal resistance in food samples in Pakistan which provides strength to this study; however, there is a limitation, too. We were unable to do molecular identification of metal resistant genes in these isolates, and it would be interesting to see the correlation of metal resistance genes and antibiotic-resistant genes in further studies.

## 5. Conclusions

Our results show that raw and processed foods, including vegetables, salad, fruits, and frozen meat, are important for disseminating ESBL genes, which pose significant consumer health risks. The highest incidence was reported for blaCTXM-1, followed by blaSHV-12, blaTEM-1, and blaCTXM-2. Strict policies and surveillance programs regarding the use of antimicrobial agents in the food and agriculture industry are urgently needed. This study also shows that heavy metal use provides a potent and previously underappreciated antibiotic resistance source.

## Figures and Tables

**Table 1:** Gram-negative isolates from processed and raw food samples (*n* = 408).

Isolates (*n*; %)	Processed Food Samples (*n* = 186)	Raw Food Samples (*n* = 222)	*p*-Value
*E. coli* (56; 13.7%)	38 (20.43%)	18 (8.11%)	<0.01
*K. pneumoniae* (76; 18.6%)	32 (17.20%)	44 (19.82%)	0.49
*E. cloacae* (58; 14.2%)	22 (11.83%)	36 (16.22%)	0.20
*C. freundii* (29; 7.1%)	16 (8.60%)	13 (5.86%)	0.28
*P. aeruginosa* (19; 4.7%)	14 (7.53%)	5 (2.25%)	0.01
*S. enteritidis* (35; 8.6%)	14 (7.53%)	21 (9.46%)	0.48
*Y. enterocolitica* (21; 5.1%)	13 (6.99%)	8 (3.60%)	0.12
*S. typhimurium* (43; 10.5%)	11 (5.91%)	32 (14.41%)	0.01
*S. marcescens* (38; 9.3%)	11 (5.91%)	27 (12.16%)	0.03
*Shigella spp.* (17; 4.2%)	9 (4.84%)	8 (3.60%)	0.53
*P. mirabilis* (16; 3.9%)	6 (3.23%)	10 (4.50%)	0.50

*p*-values were obtained from the chi-square test.

**Table 2:** Antimicrobial resistance profiles of food isolates (*n* = 408).

Antibiotics	*K. Pneumonia* (*n* = 76)	*E. Cloacae* (*n* = 58)	*E. Coli* (*n* = 56)	*S. Typhimurium* (*n* = 43)	*S. Marcescens* (*n* = 38)	*S. Enteritidis* (*n* = 35)	*C. Freundii* (*n* = 29)	*Y. Enterocolitica* (*n* = 21)	*P. Aeruginosa* (*n* = 19)	*Shigella* Spp. (*n* = 17)	*P. Mirabilis* (*n* = 16)
Ampicillin	IR	IR	41	32	IR	26	IR	IR	IR	9	11
			73.21%	74.42%		74.29%				52.94%	68.75%
Aztreonam	19	23	16	15	12	21	9	10	8	4	6
	25%	39.66%	28.57%	34.88%	31.58%	60%	31.03%	47.62%	42.11%	23.53%	37.5%
Amikacin	24	26	19	13	19	27	9	9	8	4	7
	31.58%	44.83%	33.93%	30.23%	50%	77.14%	31.03%	42.86%	42.11%	23.53%	43.75%
Gentamicin	21	24	16	12	19	27		8	7	4	7
	27.63%	41.38%	28.57%	27.91%	50%	77.14%	31.03%	38.10%	36.84%	23.53%	43.75%
Cefuroxime	28	29	19	15	IR	13	IR	11	9	7	8
	36.84%	50%	33.93%	34.88%		37.14%		52.38%	47.37%	41.18%	50%
Cefoxitin	19	IR	17	19	IR	12	IR	9	8	2	1
	25%		30.36%	44.19%		34.29%		42.86%	42.11%	11.76%	6.25%
Ceftriaxone	21	34	19	15	19	21	9	11	IR	7	3
	27.63%	58.62%	33.93%	34.88%	50%	60%	31.03%	52.38%		41.18%	18.75%
Ceftazidime	23	35	21	17	19	27	9	11	8	7	7
	30.26%	60.34%	37.50%	39.53%	50%	77.14%	31.03%	52.38%	42.11%	41.18%	43.75%
Cefotaxime	24	32	25	26	17	18	17	11	IR	9	8
	31.58%	55.17%	44.64%	60.47%	44.74%	51.43%	58.62%	52.38%		52.94%	50%
Cefepime	17	7	16	6	4	16	3	7	2		5
	22.37%	12.07%	28.57%	13.95%	10.53%	45.71%	10.34%	33.33%	10.53%	23.53%	31.25%
Ciprofloxacin	17	9	11	7	4	7	3	5	2	5	3
	22.37%	15.52%	19.64%	16.28%	10.53%	20%	10.34%	23.81%	10.53%	29.41%	18.75%
Levofloxacin	19	12	10	7	3	6	3	4	2	5	3
	25%	20.69%	17.86%	16.28%	7.89%	17.14%	10.34%	19.05%	10.53%	29.41%	18.75
Imipenem	17	6	13	6	4	7	3	5	2	3	3
	22.37%	10.34%	23.21%	13.95%	10.53%	20%	10.34%	23.81%	10.53%	17.65%	18.75%
Meropenem	1	6	12	6	4	8	3	5	2	3	3
	23.68%	10.34%	21.43%	13.95	10.53%	22.86%	10.34%	23.81%	10.53%	17.65%	18.75%
Piperacillin-Tazobactam	1	6	5	6	4		3	4	2	3	3
	15.79%	10.34%	8.93%	13.95%	10.53%	22.86%	10.34%	19.05%	10.53%	17.65%	18.75%
Colistin	1		2	1	IR	1	0	1	0	0	IR
	1.32%	1.72%	3.57%	2.33%		2.86%	0.00	4.76%	0%	0%	
Co-trimoxazole	22	21	16	15	12	14	9	10	8	4	6
	28.95%	36.21%	28.57%	34.88%	31.58%	40%	31.03%	47.62%	42.11%	23.53%	37.5%
Tigecycline	7	3	2	6	2	1	1	1	IR	1	IR
	9.21%	5.17%	3.57%	13.95%	5.26%	2.86%	3.45%	4.76%		5.88%	

IR: intrinsic resistance.

**Table 3:** Gram-negative isolates from processed and raw food samples (*n* = 408).

Isolates (*n*; %)	Processed Food (*n* = 84)	Raw Food (*n* = 89)	*p*-Value	OR (95% CI)
*K. pneumoniae* (39; 22.54%)	17 (43.59%)	22 (56.41%)	0.53	0.8 (0.39–1.63)
*E. cloacae* (16; 9.25%)	6 (37.50%)	10 (62.50%)	0.37	0.62 (0.22–1.8)
*E. coli* (36; 20.81%)	24 (66.67%)	12 (33.33%)	0.01	2.64 (1.22–5.72)
*S. typhimurium* (17; 9.83%)	8 (47.06%)	9 (52.94%)	0.93	0.96 (0.35–2.62)
*S. marcescens* (7; 4%)	2 (28.57%)	5 (71.43%)	0.29	0.42 (0.08–2.22)
*S. enteritidis* (11; 6.36%)	5 (45.45%)	6 (54.55%)	0.86	0.9 (0.26–3.06)
*C. freundii* (14; 8.1%)	3 (21.43%)	11 (78.57%)	0.03	3.71 (1.52–12.50)
*Y. enterocolitica* (5; 2.89%)	2 (40%)	3 (60%)	0.69	0.72 (0.12–4.1)
*P. aeruginosa* (14; 8.1%)	11 (78.57%)	3 (21.43%)	0.02	4.43 (1.19–12.60)
*Shigella* spp. (4; 2.31%)	3 (75%)	1 (25%)	0.273	3.34 (0.34–31.74)
*P. mirabilis* (10; 5.78%)	3 (30%)	7 (70%)	0.24	0.44 (0.11–1.78)

OR: odds ratio; CI: confidence interval; *p*-values were obtained from the chi-square test, and odds ratios were obtained by regression analysis.

**Table 4:** Distributions of ESBL *bla* gene variants (*n* = 150).

*Bla* Gene Variants(*n*, %)	*K. Pneumonia* (*n* = 36)	*E. Cloacae* (*n* = 14)	*E. Coli* (*n* = 32)	*S. Typhimurium* (*n* = 16)	*S. Marcescens* (*n* = 5)	*S. Enteritidis* (*n* = 10)	*C. Freundii* (*n* = 11)	*Y. Enterocolitica* (*n* = 3)	*P. Aeruginosa* (*n* = 12)	*Shigella* Spp. (*n* = 3)
CTX-M-1 (56, 37.33%)	17 (47.22%)	5 (35.71%)	11 (34.38%)	6 (37.5%)	1 (20%)	3 (30%)	3 (27.27%)	2 (66.67%)	4 (33.33%)	1 (33.33%)
CTX-M-2 (21, 14%)	3 (8.33%)	2 (14.29%)	6 (18.75%)	2 (12.5%)	1 (20%)	1 (10%)	2 (18.18%)	0 (0%)	2 (16.67%)	1 (33.33%)
CTX-M-9 (6, 4%)	2 (5.56%)	1 (7.14%)	1 (3.13%)	0 (0%)	0 (0%)	1 (10%)	0 (0%)	0 (0%)	0 (0%)	0 (0%)
TEM-1 (25, 16.67%)	8 (22.22%)	3 (21.43%)	6 (18.75%)	1 (6.25%)	1 (20%)	2 (20%)	2 (18.18%)	0 (0%)	2 (16.67%)	0 (0%)
TEM-135 (2, 1.33%)	1 (2.78%)	0 (0%)	0 (0%)	0 (0%)	0 (0%)	0 (0%)	1 (9.09%)	0 (0%)	0 (0%)	0 (0%)
TEM-4 (1, 0.67%)	0 (0%)	1 (7.14%)	0 (0%)	0 (0%)	0 (0%)	0 (0%)	0 (0%)	0 (0%)	0 (0%)	0 (0%)
SHV-12 (35, 23.33%)	5 (13.89%)	2 (14.29%)	7 (21.88%)	5 (31.25%)	2 (40%)	3 (30%)	3 (27.27%)	1 (33.33%)	3 (25%)	1 (33.33%)
SHV-11 (4, 2.67%)	0 (0%)	0 (0%)	1 (3.13%)	2 (12.5%)	0 (0%)	0 (0%)	0 (0%)	0 (0%)	1 (8.33%)	0 (0%)

## Data Availability

Data are contained within the article.
